# Sensory neuron activation from topical treatments modulates the sensorial perception of human skin

**DOI:** 10.1093/pnasnexus/pgad292

**Published:** 2023-09-26

**Authors:** Ross Bennett-Kennett, Joseph Pace, Barbara Lynch, Yegor Domanov, Gustavo S Luengo, Anne Potter, Reinhold H Dauskardt

**Affiliations:** Department of Materials Science and Engineering, Stanford University, Stanford, CA 94305, USA; Department of Mechanical Engineering, Stanford University, Stanford, CA 94305, USA; L’Oréal Research and Innovation, Aulnay-sous-Bois 93601, France; L’Oréal Research and Innovation, Aulnay-sous-Bois 93601, France; L’Oréal Research and Innovation, Aulnay-sous-Bois 93601, France; L’Oréal Research and Innovation, Aulnay-sous-Bois 93601, France; Department of Materials Science and Engineering, Stanford University, Stanford, CA 94305, USA

**Keywords:** perception, skin biomechanics, mechanoreceptors

## Abstract

Neural signaling of skin sensory perception from topical treatments is often reported in subjective terms such as a sensation of skin “tightness” after using a cleanser or “softness” after applying a moisturizer. However, the mechanism whereby cutaneous mechanoreceptors and corresponding sensory neurons are activated giving rise to these perceptions has not been established. Here, we provide a quantitative approach that couples in vitro biomechanical testing and detailed computational neural stimulation modeling along with a comprehensive in vivo self-assessment survey to demonstrate how cutaneous biomechanical changes in response to treatments are involved in the sensorial perception of the human skin. Strong correlations are identified between reported perception up to 12 hours post treatment and changes in the computed neural stimulation from mechanoreceptors residing deep under the skin surface. The study reveals a quantitative framework for understanding the biomechanical neural activation mechanism and the subjective perception by individuals.

**Significance Statement**

This work makes a significant contribution to describing the neural mechanisms responsible for human skin sensorial perception, adding to the body of knowledge of neuroscience. The combination of ex vivo biomechanical measurements, in silico computational models, and in vivo self-assessments of skin sensorial perception employed in this work establishes an improved framework for studying the effects of novel topical formulations relevant to a wide range of industries.

## Introduction

**Fig. 1.**
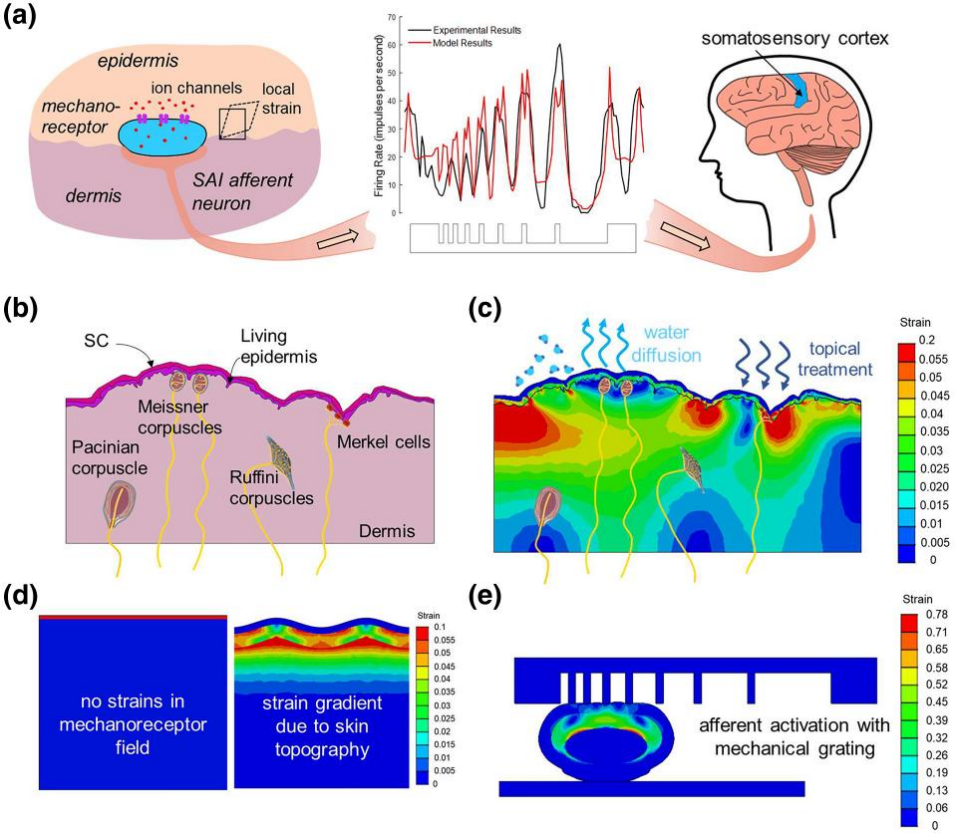
Describing sensory neuron activity from biomechanical changes of skin. a) Schematic of SA I neural signals from the skin to the somatosensory cortex of the brain. A Merkel cell at the epidermal-dermal junction with its mechanically gated ion channels is depicted. Firing rates measured from indenting primate fingertips are shown with the corresponding computed firing rates based on FE simulations. b) Depiction of the cutaneous mechanoreceptors in the epidermis and dermis of human skin. c) Representation of the distribution of strains that develop from SC drying. SC drying causes strains to develop at the depths of cutaneous mechanoreceptors. d) The flat SC (left) is highlighted in a skin section model to illustrate that SC drying that results in SC stresses of 3 MPa does not cause strains to develop in underlying skin. Strains develop at the depths where mechanoreceptors reside in a skin section model with SC topography (right). e) Strains calculated from a FE model replicating one indentation of the mechanical grating in primate fingertip skin.

Receptors in human skin transduce mechanical (1–4), thermal (5), and chemical (6) stimuli through a complex system of sensory neurons and neural pathways in the somatosensory system. Perception of changes in the skin mechanical environment in response to touch (7), dehydrating sun exposures (8), or cosmetic treatments (9) is mediated by cutaneous mechanoreceptors (1) that modulate signals relayed by peripheral neurons to the central nervous system (CNS). An example shown schematically in Fig. 1a involves an oval-shaped Merkel cell mechanoreceptor and its associated slowly adapting type I (SAI) neuron responsible for light touch sensations.

The sensorial skin perception after application of a topical treatment involves the interpretation of neural signals from mechanoreceptors and associated sensory afferent neurons by the CNS (Fig. 1b). The treatments induce biomechanical changes in the skin that activate signaling. Cleansers and moisturizers are topical treatments that interact with the outer stratum corneum (SC) skin layer to clean or hydrate. Their formulation involving many components described in more detail below affects the development of mechanical stress in the SC as it dries in the hours following the application. The SC stress induces mechanical strain below the skin surface, as shown by the color variations in Fig. 1c, providing a clue for the activation of sensory neurons.

Changes in the mechanical state of the SC after topical skin treatment include contraction due to both water loss and intercellular lipid stripping (10) and swelling from an increase in water content and diffusion of treatment components into the SC (11). These alter the subjective judgment of skin comfort. While the effects of cleansers and moisturizing treatments on SC mechanical properties and stresses can be quantified with in vitro measurements, their in vivo effects on skin sensory perception are subjectively reported in carefully constructed product testing surveys with high statistical confidence (12). Subjective terms such as a sensation of skin “tightness” after using a harsh cleanser or a sensation of skin “softness,” “moisturization,” or “absence of skin tightness” after applying an efficacious moisturizer are used to evaluate treatments. However, the detailed mechanism whereby sensory neurons are activated by changes in the mechanical state of the SC to carry signals to the CNS, where a perception is formed, has not been established.

The mechanism must address the fundamental question of how a topically applied chemical formulation results in a mechanical stimulus for mechanoreceptors that influence the transmission of action potentials in corresponding afferent neurons (Fig. 1a) (13). Note that this work does not treat known biochemical signaling that may be associated with molecules like sodium lauryl sulfate (14, 15) or phenol (16) that act as irritants in some exfoliating and cleansing formulations. These lead to immediate and sometimes chronic perceptions of discomfort including itching, stinging, and burning that are related to inflammatory responses including increased cytokine production (17). Other forms of skin perception related to temperature changes, such as the perception of cold or hot surfaces and skin “wetness” (18), are not considered either as these are mediated by thermally sensitive nonspecialized free nerve endings (5). Rather, this work details the biomechanical mechanism whereby the application of topical treatments, such as cleansers or moisturizers, changes the mechanical state of the topmost SC layer of human skin that subsequently changes the mechanical environment of the underlying skin layers leading to the activation of afferent neurons and a perception related to skin comfort.

Mechanoreceptors serve to modulate action potentials at embedded nerve endings, and several types of receptors initially appear near the dermal-epidermal junction (DEJ) (Fig. 1b). Mechanoreceptors are characterized in terms of a specific morphology that contributes to their function (1, 19, 20). The receptors are associated with either fast-adapting neurons that generate action potentials at the onset and offset of mechanical stimuli or slowly adapting neurons that continue to fire for the duration of a sustained stimulus's presence. As the subjective perception of mechanical changes in the SC is the result of sustained stresses that form in the SC over long-time scales of several hours, the fast-adapting neurons associated with Meissner corpuscles and Pacinian corpuscles are not expected to contribute to the sustained perception of SC changes manifested as skin tightness. They are sensitive to high-frequency events as described in a recent simulation study involving both fast and slowly adapting neurons (21). Further, Pacinian corpuscles are not found in human facial skin (22).

Though Ruffini endings which are located in the dermal layer (23) are associated with slowly adapting type II (SAII) neurons, there is only limited evidence that SAII afferent activity contributes to conscious perception (24). Multiple studies suggest that stimulation of single SAII afferents largely is unperceived and that SAII afferents contribute to proprioception (25–27).

Merkel cells and the associated slowly adapting type I (SAI) neurons have been commonly characterized to detect sustained skin indentation (1). SAI neurons are also known to be activated by stretching of the skin (28, 29), a similar mechanical situation to stresses caused by mechanical changes in the SC. The Merkel cell-SAI neuron complex is therefore of particular interest to this study as the most likely receptor to explain the sustained perception of biomechanical changes in the SC upon activation. As is the case with perception of other mechanical stimuli, it is likely that more than one receptor is affected in response to biomechanical changes in the SC as previously demonstrated for natural stimuli (30); however, the strongest case for a relation between sustained skin tightness perception and neural activity can be made for SAI afferents, which are the afferents of focus in this work. C afferent fibers which can be associated with the sense of pleasant touch (31) and demonstrate intermediate adaptation rates between typical fast and slowly adapting neurons (32) are not considered in this study. For a review of low-threshold cutaneous mechanoreceptors, see references (1–4).

Changes in the SC outermost layer of human skin are particularly important for explaining the effects of treatments on skin perception. Ranging in thickness from ∼10 to 30 µm depending on anatomical site (33), the SC consists of a network of terminally differentiated and interconnected cornified cells (corneocytes) embedded in a lipid matrix (34) and makes only a small contribution to the total skin thickness which can be up to ∼1.5 mm in the human face (35). However, the SC has markedly different mechanical properties compared to the underlying skin layers, exhibiting a high elastic stiffness (36) and linear elastic stress-strain behavior to large physiological strains. SC mechanical properties also change significantly with hydration, with moisturizing treatments, or from damaging processes such as lipid extraction, exposure to harsh cleansing treatments, or UV solar exposure that leads to excess drying (8, 37–39). It is therefore surprising that not much attention has been given to the role of SC changes in skin sensory perception and even more surprising that the SC is often neglected in skin biomechanical models (40–46).

Skin treatments are well known to affect SC components such as water content, lipid content, and lipid order (47–53). As the water content increases with a moisturizing treatment or high relative humidity in the environment, the SC layer swells and the elastic modulus decreases from ∼100 to 200 MPa in ambient conditions to values as low as ∼2 MPa (11). Molecules in the treatment aside from water may diffuse into the SC and contribute to SC expansion (54). Alternatively, water loss and lipid loss related to exposure to a harsh cleanser or a dry environment lead to SC contraction and stiffening to an elastic modulus that can exceed 250 MPa (38). As the SC is bound to underlying skin, SC contraction is manifested by out-of-plane SC thickness changes and compensated by in-plane elastic strains. In-plane biaxial SC stresses associated with the elastic strains are measured via in vitro experiments and can exceed 4 MPa (38, 54–57). These stresses are supported by mechanical forces in the SC (58) and are significantly higher than the inherent or exogenous full thickness skin stresses of ∼15 kPa and even endogenous stresses related to physiological motion reported to be as high as ∼60 kPa depending on the body location (59).

Moisturizing treatments employ several mechanisms for preventing water loss and reducing the perception of stress or tightness in the skin. Humectant species in moisturizers act as hydrophilic molecules that serve to raise the energy barrier for water loss. Occlusives form a physical barrier between the SC and a dry external environment. Finally, emollients serve to replace lost volume, which counteracts stress development from water loss. Keratolytic actives may directly plasticize keratin and other proteins. One or all of these chemical approaches may be implemented in a complex moisturizer formulation (54, 55, 60, 61). The treatments used in this study include up to 30 components each of which can act on the skin to reduce or induce stress. Quantitative prediction of skin perception simply based on the composition of a treatment's components and formulation does not currently exist.

The function of cleanser formulations is to remove oils and dirt on the skin surface. To accomplish this, cleanser formulations often include harsh surfactants that may strip the skin of mobile lipid content (56, 57), which can be highly disruptive of the skin's natural water barrier function (34). Disruption of the SC barrier leads to increased water loss and an associated increase in tensile stresses in the SC which heightens the perception of skin tightness. Innovations are frequently sought to develop milder cleansers that respect the skin barrier.

Here, we provide a quantitative methodology for evaluating and predicting the perception of skin “tightness” many hours after application of cleanser or moisturizing treatments. We focus on the magnitude of the in vitro SC stresses that develop under controlled drying conditions and demonstrate that these stresses are the cause of mechanical strains in underlying skin tissue that stimulate mechanoreceptors and activate neural responses perceived as skin tightness. Based on SAI afferent neuron firing rates measured in primate fingertips in response to mechanical stimulation, we develop a general relation between strain state at the location of Merkel cells and SAI firing rates which we employ to predict neural stimulation in human skin caused by SC drying following the application of topical treatments.

Controlled human sensorial perception assessment surveys involving thousands of participants in a multiyear study provide statistically significant quantitative scores to accurately discriminate perceived skin tightness following the cleanser and moisturizing treatments.

Our approach couples in vitro mechanical testing and detailed computational neural stimulation modeling along with a comprehensive in vivo human sensorial perception assessment participant survey to demonstrate how cutaneous biomechanical changes in response to topical treatments are involved in the sensorial perception of human skin. Strong correlations are identified between reported perception up to 12 hours post treatment and computed SAI neuron activity below the skin surface. The approach represents a quantitative framework for understanding the biomechanical mechanism whereby SAI sensory neurons are activated in response to topical skin treatments and subjectively perceived by individuals.

### Human skin model

We simulated the effect of in vivo SC drying via the finite element (FE) method (Abaqus/CAE/Standard 2019, Dassault Systèmes Simulia Corp., Johnston, RI, USA) on full-thickness human skin models. Geometries representative of a diversity of skin surface topographies were reproduced from histology images of cheek, forehead, and abdomen cross-sections found in the literature (45, 62), and the outlines of the skin layers were traced. Two-dimensional FE skin section models were created with a dense mesh of linear triangular plane strain elements. The plane strain assumption in the model treats the skin section as if the cross-section geometry is constant out of the plane, which makes it possible to clearly model the effect of skin surface topography on deformations in underlying skin layers. In the histology images, the SC, living epidermis, and dermis were distinguishable, and the relative thicknesses of the layers were consistent with what is reported in literature (35, 63–66).

We modeled SC drying as a dilatory contraction (uniform volume decrease) of the SC layer. The left and right edges of the models were constrained horizontally, and the lower edge was constrained vertically. From separate simulations of the in vitro stress measurement setup, we observed that a dilatory SC contraction of 1.5% resulted in similar in-plane SC stress measured after drying an untreated SC sample. By comparison, the effects of the moisturizer and cleanser treatments measured in vitro resulted in SC contractions less or greater than 1.5%, after the treatments were applied. The SC was modeled as an isotropic, linear elastic material with a modulus of elasticity (*E*_SC_) of 200 MPa and a Poisson's ratio (ν_SC_) of 0.43. The living epidermis and dermis were modeled as isotropic Neo-Hookean materials with initial moduli of elasticity of 500 kPa and 50 kPa, respectively, and a Poisson's ratio of 0.48 and the simplified reduced-polynomial strain-energy function to obtain the Neo-Hookean form involving coefficients of *c*_10_ = 84,459.5 and *d*_1_ = 4.8e^−07^ for the living epidermis and *c*_10_ = 8,445.95 and *d*_1_ = 4.8e^−06^ for the dermis. We note that while *E*_SC_ is hydration sensitive and significantly lower on treatment application, only the final *E*_SC_ after drying was used in the simulations as the initial *E*_SC_ value had no effect on the final predicted skin strain state.

Roughness of the skin surface plays a significant role in the distribution of strains in the living epidermis and dermis from contraction of the SC. We note, imperatively, that in the absence of surface roughness, SC contraction from drying does not lead to any strain in the underlying layers (Fig. 1d, left). Indeed, well-known thin film mechanics reveal that for a flat SC layer, there are no stresses at the SC and epidermis interface. Also, there is no vertical component of stress acting on the sub-SC layers of the epidermis as the SC has a mechanically free top surface. However, with skin topography, in-plane stresses within the SC frame of reference result in shear stress at the SC and epidermis interface and vertical stress components acting on the underlying layers of the epidermis where associated strain distributions result. Topographical features of the skin surface result in the propagation of strain to depths of mechanoreceptors, thereby enabling the perception of skin discomfort from SC drying (Fig. 1d, right).

Computed strains from the moisturizer and cleanser treatment simulations were used to predict the SAI neuron firing rate based on a relation between strain and firing rate that we describe below. The estimated firing rate was computed in each element along the epidermal side of the DEJ where SAI neurons terminate in Merkel cells. The mean value of these firing rates was used to examine correlations with the human sensorial perception assessment study skin tightness perception scores.

### SAI afferent neuron firing rate model

To develop a general relation between strain at the location of Merkel cells and SAI firing rates, we relied on reported SAI afferent neuron firing rates in primate fingertips from mechanically indenting the fingertip using an aperiodic grating with different points along the grating located in the neuron's receptive field for each indentation (67). The firing rates reported are based on neural activity measured 600–950 ms after the onset of each indentation, representing the more stable firing rate during static indentation.

Importantly, we note that we seek a firing rate relation dependent exclusively on the *strain* state and not the *stress* state as sometimes employed (40, 43). The reason is that we are concerned with displacement-induced activation directly correlated with the strain state, and while there is a connection to mechanical stresses, these relax in a viscoelastic tissue potentially leading to decreasing firing rates over short time scales (68); however, these are not relevant in the present work.

We computed the expected strains in the primate fingertip from the indentations by FE simulation (Abaqus, *Ibid*) (Fig. 1e). We created a more detailed primate finger cross-sectional model to bolster the accuracy of the relationship between the measured firing rates and mechanical strain compared to previous approaches (40, 43, 69). We incorporated multiple skin layers—SC, living epidermis, dermis, and hypodermis—and the distal phalanx. Specifically, modeling the SC as a distinct layer from the rest of the epidermis was not included in previously reported models of the primate fingertip indentation experiment but clearly crucial for the present study. Inverse FE analysis was used to assign more accurate mechanical properties of the skin layers based on fingertip skin surface deflection data reported from indentation by a wedge (70).

We assumed that a SAI afferent was located midway between the lateral and medial edges of the distal fingertip and evaluated strain in an element on the epidermal side of the DEJ. The optimal relation between computed strains and reported firing rate was computed by minimization of a multivariable function of local strain components. Strong correlations between measured and predicted firing rates were obtained for several strain types. We calibrated the model using the effective strain, as it is a strain invariant describing the deviatoric strain state of a material. The relation between the effective (von Mises) strain and the firing rate was found to be:


(1) $$R_{\mathrm{predicted}}=c_{1}(\varepsilon -c_{2}),$$

where *R*_predicted_ is the firing rate of the SAI afferent, *ε* represents the effective strain, and *c*_2_ is the effective strain threshold which was constrained to be nonnegative. A best fit was found with *c*_1_ = 179.1 and *c*_2_ = 8.1e^−9^. The goodness of fit between firing rates predicted by Eq. 1 and strains measured experimentally was evaluated by the fractional sum of squares (FSS) (69):


(2) $$\mathrm{FSS}=1-\frac{\sum_{i}\;{\left(R_{i,\mathrm{experiment}}-R_{i,\mathrm{predicted}}\right)}^{2}}{\sum_{i}\;R_{i,\mathrm{experiment}}^{2}},$$

where *R*_i, experiment_ is the measured firing rate for a given indentation in the experiment and *R*_i, predicted_ refers to the predicted firing rate for the corresponding indentation from Eq. 1. The FSS between measured and predicted firing rates was 0.87. An example of the fidelity of the predicted firing rate model compared to one of many of the measured firing rates is shown in Fig. 1a. Additional features in the predicted firing rates including the appearance of double peaks for certain indentation features are ascribed to the greater fidelity of the model to distinguish more subtle variations of the local strain fields compared to the experimental measurement. We note that Eq. 1 represents a generalized firing rate dependence on the effective strain state, independent of the manner in which the strains are induced, e.g. via mechanical probing as in the primate studies or contraction of the SC relevant in the present work.

## Materials and methods

### Human sensorial perception assessment

Our human skin sensorial perception assessment focused on nine moisturizers (Tables 1 and 2) that were assessed by two groups of 2,000 women participants, ranging in age from 20 to 60 years, in spring 2015 and spring 2016 in France. Extreme skin types were excluded. Each moisturizer was assessed by ∼160 women. Six cleansers (Tables 1 and 2) were assessed by 120 women each in China in spring 2017. The products were evaluated after 1 week of home use by self-assessment using questionnaires that assessed many aspects of skin perception related to facial skincare (e.g. moisturization effect, freshness, radiance, texture, and application) from multiple perspectives (e.g. tactile, visual, and olfactory).

**Table 1.** Components of each of the studied treatments are provided.

Designation	Commercial Name	Water	Alcohol	Polyol/humectants	Oils/fats	Silicones	Emulsifiers/surfactants	Simulation input: % contraction
M-A	Submica Light	57.7%	9.3%	15%	8%	0%	3%	1.33
M-B	Skin Perfector	61.1%	8%	9%	5%	12%	1.2%	1.29
M-C	Emulsified Gel Amps	78.9%	5.0%	12%	0%	1.76%	1%	1.24
M-D	Shine Trap Moist	71.7%	0%	10%	11%	4%	0%	1.21
M-E	Lipogel	64.2%	0%	7%	0.42%	20%	5%	1.20
M-F	Fluidonov	65.4%	4.7%	15%	6%	1.44%	0%	1.11
M-G	Memory Shape Risocast	51.9%	3.0%	12%	18%	11%	0%	1.04
M-H	Hydra Regulator	68.4%	0%	12%	13%	0%	1.92%	0.94
M-I	Socle Naturel DPGP	65.5%	1.9%	7%	18%	0%	3%	0.91
C-A	Creamy Fresh							2.41
C-B	OAP White							2.23
C-C	VA Soap							2.22
C-D	Starch Gel G3							1.53
C-E	Amilite Cream							1.43
C-F	Amilite Gel							1.41

**Table 2.** Composition of each of the studied treatments.

M-A	Water, preservatives, glycols, sodium hyaluronate, glycerin, gum, mica, polyglyceryl-3 dicitrate/stearate, dicaprylyl ether, wax, titanium dioxide, fragrance
M-B	Water, preservatives, glycols, salicylic acid, capryloyl glycine, ammonium polyacryloyldimethyl taurate, sodium polyacrylate, gum, glycerin, peg, oil, dimethicone, isostearyl neopentanoate, silica silylate, fragrance, capryloyl salicylic acid
M-C	Water, preservatives, glycols, glycerin, sodium hyaluronate, gums, ammonium polyacryloyldimethyl taurate, dimethicone, fragrance
M-D	Alcohols, glucoside, behenyl behenate, dimethicone, isononyl isononanoate, squalane, capryloyl, salicylic acid, water, preservatives, glycerin, sodium taurate (and) polysorbate (and) sorbitan oleate fragrance
M-E	Water, preservative, glycols, glyceryl stearate, glycerin, titanium dioxide, dimethicone, gum, copolymer, fragrance
M-F	Wax, peg, cetyl esters, xyloside, dicaprylyl ether, dimethicone, isopropyl palmitate, water, preservatives, glycols, copolymer, glycerin, nylon, silica, fragrance
M-G	Water, preservative, glycerin, sodium hyaluronate, glycols, gum, vegetal oils, silicones, capryloyl salicylic acid, polymer, tocopherol, ascorbyl glucoside, fruit extracts, silica, fragrance
M-H	Water, preservative, glycols, glycerin, sodium stearoyl glutamate, cetyl esters, wax, capryloyl salicylic acid, dicaprylyl ether, coco-caprylate/caprate, tocopherol, sodium hyaluronate, starch, dicaprylyl ether, fragrance, copolymer
M-I	Salicylic acid, glycols, stearates, stearic acid, triglyceride, butter, fragrance, water, glycerin, tocopherol, polymer, gum, starch, preservative
C-A	Water, preservatives, glycols, titanium dioxide, hydroxypropyl methylcellulose, sorbitol, kaolin, glycol distearate, coco-betaine, lauric acid, myristic acid, stearic acid, polyquaternium, fruit extract, fragrance
C-B	Stearic acid, palmitic acid, myristic acid, lauric acid, glyceryl distearate (and) glyceryl stearate, water, preservatives, glycerin, peg, guar, salicylic acid, perlite, fragrance
C-C	Water, preservatives, glycerin, stearic acid, palmitic acid, myristic acid, lauric acid, glyceryl distearate (and) glyceryl stearate, salicylic acid, kaolin, peg, perlite, fragrance
C-D	Water, preservatives, glycols, sodium lauroyl sarcosinate, ethylhexylglycerin, cocamide mea, glycol distearate, acrylates copolymer, acrylates/steareth-20 methacrylate copolymer, lauric acid, starch, coco-betaine, peg, fragrance
C-E	Glycerin, carbomer, water, preservatives, glycols, polyquaternium, sodium cocoyl glycinate, glycol distearate, stearic acid, fragrance
C-F	Water, preservatives, glycols, glycerin, acrylates copolymer, acrylates/steareth-20 methacrylate copolymer, salicylic acid, sodium cocoyl glycinate, glycol distearate, titanium dioxide, coco-betaine, peg, fragrance

The questionnaires were administered in the French language for the moisturizers study and in the Chinese language for the cleanser study. Study volunteers were asked to comment on any discomfort on the cheek and forehead and were not informed of any particular interest such as skin tightness to prevent any bias in the responses. The questionnaire was administered using a mobile phone app, and responses were recorded at the end of the day following application of the treatment in the morning. With regard to tightness, the volunteers were asked to rank their level of agreement with a statement—“My skin feels tight” for the moisturizers survey in France and “After rinsing and wipe-off, would you say your skin feels tight?” for the cleansers questionnaire in China—on a five-point scale ranging from strongly disagree to strongly agree. The percentage of women disagreeing with the statements was considered the “Skin Tightness Perception Score.” That is, a higher skin tightness perception score represents a larger proportion of women that did not experience significant tightness from the product. The skin tightness perception scores are thus derived from percentages of responses given in the assessment and, as such, do not have an associated standard error.

### SC preparation

Full-thickness samples of human abdominal skin from Caucasian females between 30 and 90 years old were obtained through the National Disease Research Interchange (NDRI). The samples were stored at −80°C until processing. After removing subcutaneous tissue, the skin was soaked in 35°C water for 10 minutes and then 60°C water for 1 minute. The epidermis and dermis were then separated using a flat-tipped spatula. Epidermal tissue was removed from the SC by floating the tissue in a trypsin enzymatic digest solution [0.1% (wt/wt) in 0.05 M, pH 8.3 Tris buffer] at 35°C for 180 minutes. The SC was rinsed and dried on filter paper (medium flow, grade 995 filter paper; Whatman) and then removed to be stored in a low-humidity chamber (∼10–20% RH) at an ambient temperature of ∼18–23°C. All study procedures are previously institutional review board approved, and the SC isolation was consistent with extensive prior works that demonstrated no adverse effects on SC properties (39).

### Drying stress profiles

**Fig. 2.**
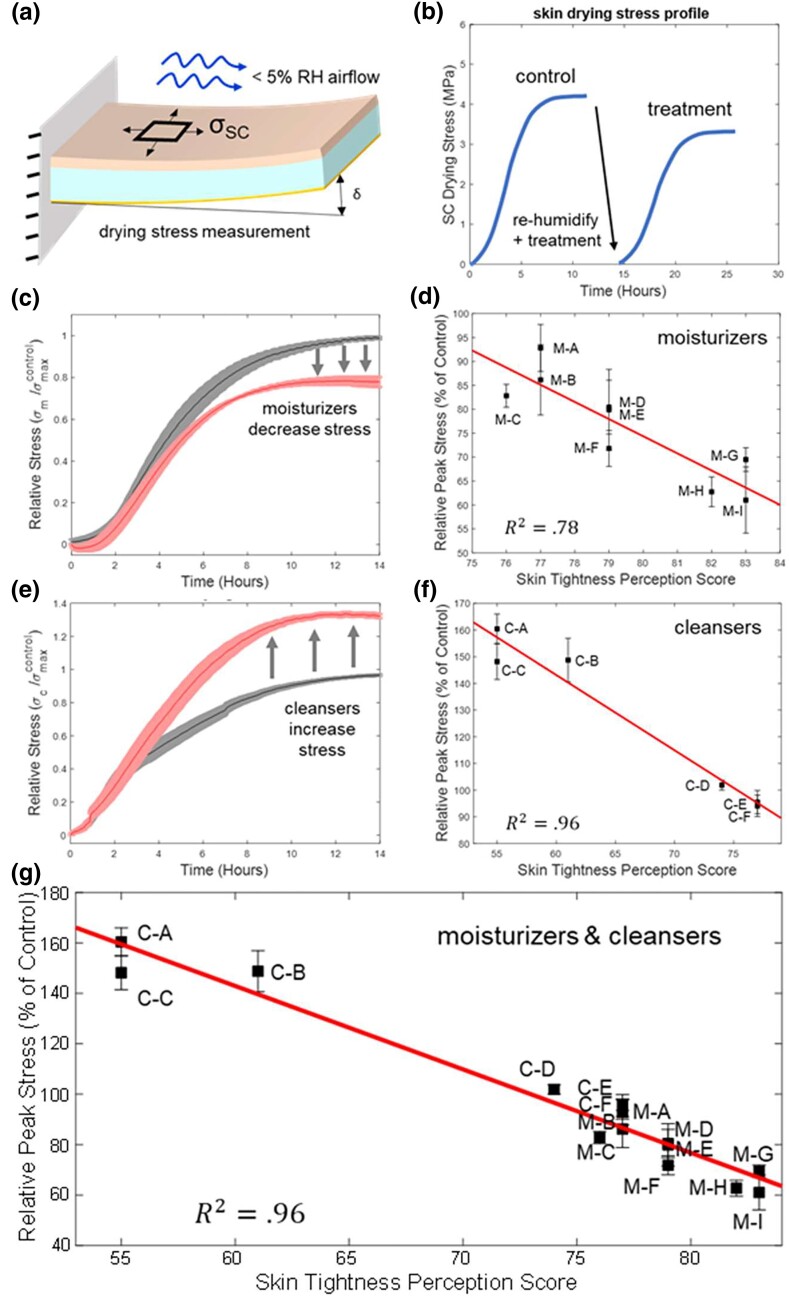
Sc drying stress measurements. a) Setup of the cantilever system for measuring SC drying stresses. b) Representation of SC drying stresses on control sample and treated sample. c) Representative reduction in relative SC stress from an effective moisturizer. d) Relative peak stresses for each moisturizing treatment against study skin tightness perception scores. e) Representative increase in relative SC stress after applying a harsh cleanser. f) Relative peak stresses for each cleanser versus skin tightness perception scores. g) Relative peak stresses for both moisturizers and cleansers versus skin tightness perception score. The vertical scatter bands represent the standard error in relative peak stress measurements for each treatment. Presented data are from *N* = 5 tests per moisturizer and *N* = 4 tests per cleanser.

Hydrated SC was adhered to a 9-mm × 35-mm × 145-μm-thick borosilicate glass beam coated on the other side with conductive Cr/Au (35 Å/465 Å). The SC adhered to the glass without the application of an adhesive. The beam was clamped on one end and the SC allowed to dry in a chamber with a controlled temperature and humidity air flow. Stresses in the SC caused the beam to deflect, which was detected by a capacitive sensor with submicron precision (Fig. 2a). Curvature of the substrate, which is derived from the amount of substrate deflection, was used to calculate SC film stresses according to Stoney's equation as we have reported for SC (71).

For moisturizer studies, untreated SC specimens were first dried as described above providing a control SC stress curve for the tissue sample and subsequent moisturizer test. The control specimens were then transferred to a 100% RH chamber for 2 hours where the SC fully rehydrated and the stresses returned to zero. Next, 2 mg/cm^2^ of the moisturizing treatment was uniformly applied on the SC surface with the side of a pipet or dowel. The SC and glass were returned to the cantilever test system where the SC stresses were measured for 10–15 hours.

For cleanser studies, a SC sample was placed in a 3:1 solution of Evian water (Danone, France) to cleanser for 2 hours. The SC was then rinsed for 30 seconds in Evian water four times before being adhered to a glass beam. To control for the effect of the cleanser, a separate SC sample was placed in pure Evian water for 2 hours and then similarly rinsed four times before being adhered to a glass beam. The treated and control samples were placed in the dry box cantilever system, and SC stresses were measured for 10–15 hours.

Stoney's equation (72) was used to relate SC drying stress, $\;\sigma_{\mathrm{SC}}$, and elastic displacement of the glass cantilever, δ


(3) $$\sigma_{\mathrm{SC}}=\left(\frac{E_{\mathrm{sub}}}{1-\nu_{\mathrm{sub}}}\right)\left(\frac{h_{\mathrm{sub}}^{2}}{3h_{\mathrm{SC}}\;*\;L^{2}}\right)\delta ,$$

where *E*_sub_, *ν*_sub_, and *h*_sub_ are the Young's modulus, Poisson's ratio, and thickness of the glass substrate, respectively. The initial and final thickness values of the SC, *h*_SC_, were measured with a digital micrometer and used for calculating $\sigma_{\mathrm{SC}}$. The SC is assumed to be a thin film compared to the substrate since the product of the film biaxial modulus and thickness are ≤1/80th of the equivalent product for the substrate. This assumption is useful since the elastic properties of the SC film are not required for measuring SC stress (71).

Each biomechanical characterization included a control drying curve, rehydration to zero stress, followed by the drying curve after treatment. At least five repeats (control plus treatment) on three different donor tissue samples were conducted for the moisturizers and four repeats on two tissue donor samples for the cleansers. The standard error for all testing resulted in generally less than 5% variation across all treatments and tissue samples assessed. Moisturizer results were obtained from three separate tissue donors for a total of *N* = 5 tests per moisturizer, and cleanser results were obtained from two separate tissue donors for a total of *N* = 4 tests per cleanser. Due to the self-normalizing nature of this test, a grand average of samples was computed across donors.

### Air flow and drying conditions

The drying conditions employed regulated 5% RH dry air at 27°C flowing at a rate of 6 L/min into the ∼2.7-L specimen chamber (Fig. 2a). These air flow conditions were maintained through a humidity control gas flow system to control individually the humidity and flow rate of five lines of air in parallel into five specimen chambers.

## Results

Control tissue peak drying stress profiles exhibiting stress plateaus with $\sigma_{\mathrm{SC}}$ ∼ 3–6 MPa dependent on the donor were measured consistent with previous literature (Fig. 2b) (9, 36, 38, 73). Subsequently, flowing humid air rehydrated the SC and $\sigma_{\mathrm{SC}}$ returned to zero. After application of a moisturizer, the drying stress profile was reduced to lower values as indicated in Fig. 2b, while the application of a cleanser was found to elevate stresses. Consistent trends in drying stresses were observed within and between the different donors. To control for biovariability across donors, statistically consistent and significant results were obtained by normalizing the results to the control peak stress values obtained for each donor as shown for a representative moisturizer and cleanser in Fig. 2c and e, respectively. The best performing moisturizing treatments were found to reduce the peak SC drying stress by ∼40% while the harshest cleanser treatment was found to increase SC drying stress by ∼60% compared to control.

The human sensorial perception assessment results indicate three groups of three moisturizer treatments that were consistently perceived as “top,” “mid,” and “flop” performers according to study participant responses. Similarly, the six cleanser treatments are separated into “harsh” and “mild” groups based on study participant responses. Harsh cleansers were perceived consistently negatively while mild cleansers had neutral or favorable perceptions. Tightness perception scores represent the percentage of participant that reported feeling either no skin tightness or very little skin tightness from use of the treatment. Strong correlations with high statistical relevance were apparent between the measured ex vivo peak stress changes and the skin tightness perception scores for both moisturizers and cleansers shown in Fig. 2d and f, respectively. Statistical *R*-squared coefficient of determination values are included in the figures. Furthermore, when plotted together (Fig. 2g), these results continue to show a strong trend in the form of a “master perception curve” with even higher statistical *R*-squared values of ∼0.96 that continues between the moisturizer and cleanser treatments.

**Fig. 3.**
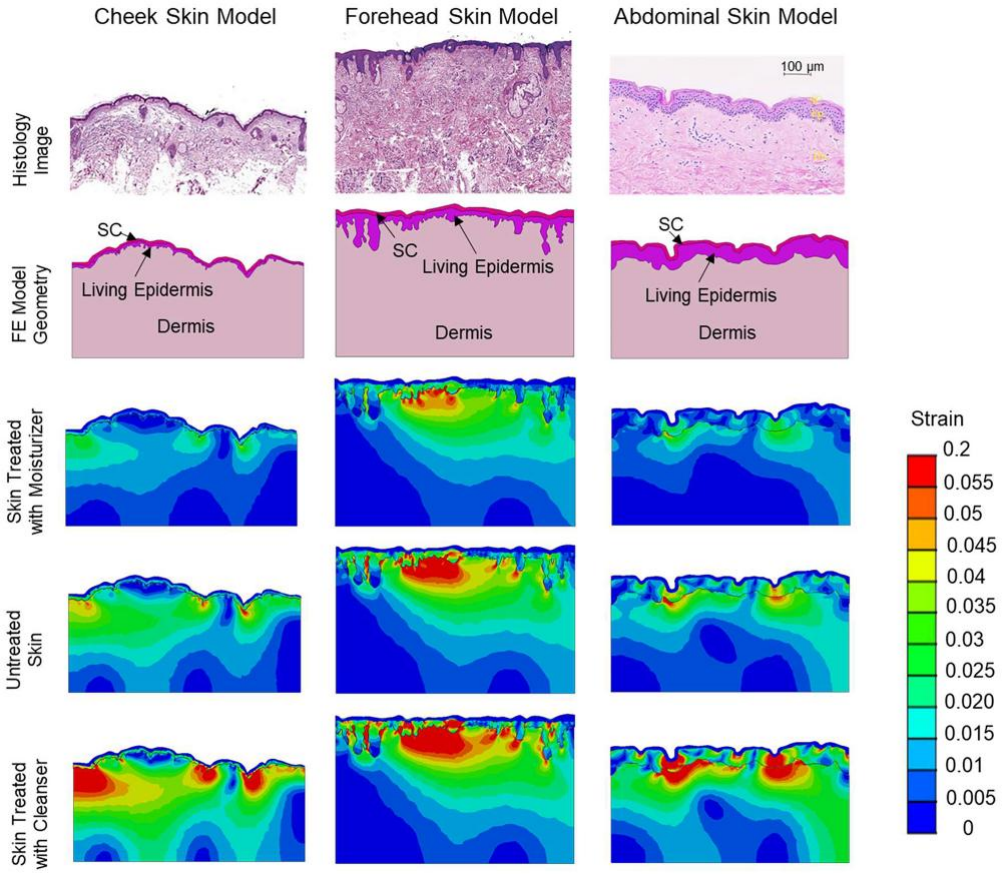
Skin section finite element modeling. Histology images reproduced from literature (43, 60) from cheek, abdomen, and forehead sections are shown in the top row. The second row displays the corresponding geometries used in the FE models. Computed principal strain distributions after drying are shown for each geometry for skin that was treated with an effective moisturizer, untreated skin, and skin that was treated with a harsh cleanser in the third, fourth, and fifth rows.

The histological cross-sections taken from published studies of the human cheek, forehead, and abdominal skin together with their traced sections where the SC, living epidermis, and dermis were distinguished and could be meshed for the FE human skin model are shown in Fig. 3 (top two rows). After computing the SC contraction related to the drying stress profiles for control and treated skin, the resulting FE simulations of the principal strain fields that develop in the underlying skin layers in response to treatments are shown in Fig. 3 (bottom three rows). The significantly decreased strain fields from control for one of the moisturizers (M-I) is clearly apparent together with the significantly elevated strains related to one of the cleaners (C-A). These trends were consistent for the three different skin locations.

**Fig. 4.**
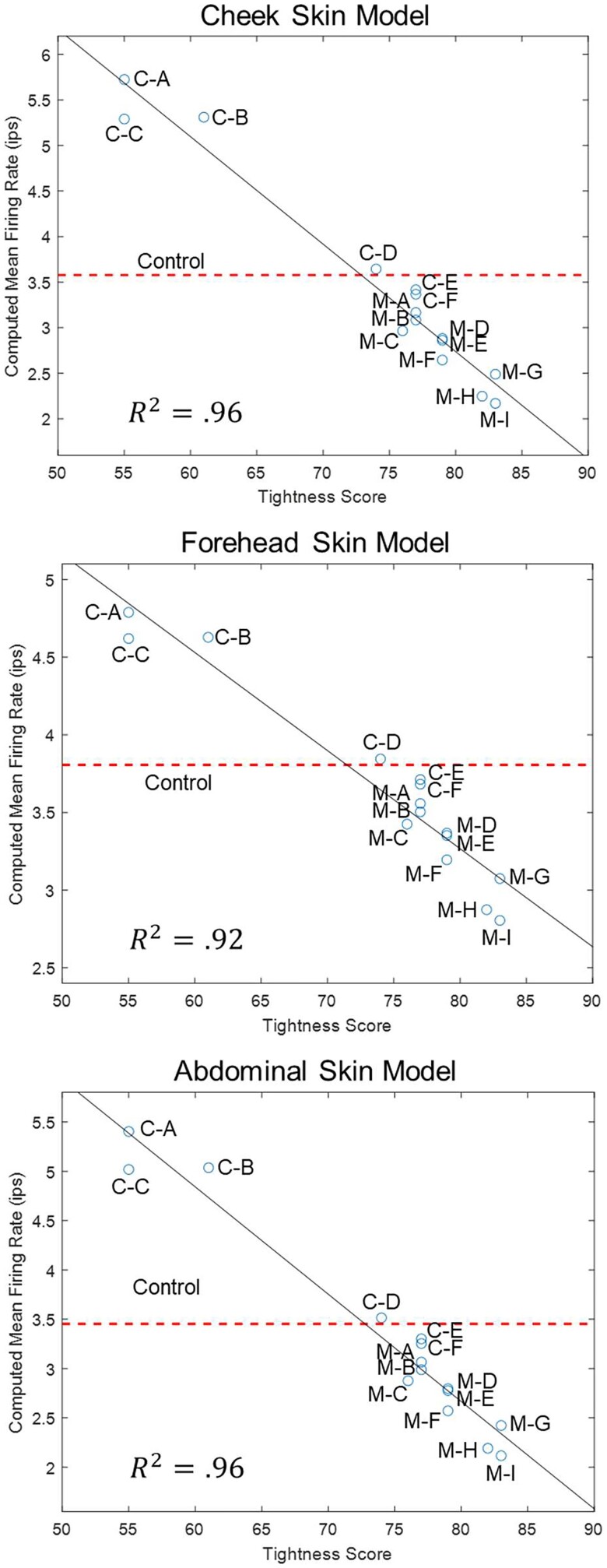
Computed firing rates from each topical treatment. The mean firing rate computed among elements along the epidermal-dermal boundary is plotted against perceived skin tightness scores for each topical treatment applied to each skin model geometry.

Using the strain fields from elements in the epidermis near the DEJ, the mean SAI firing rates from Merkel cells in these locations were computed for each treatment. The computed firing rates correlate linearly with the tightness perception scores for all skin treatments in all three skin model geometries with *R*^2^ values of 0.96, 0.92, and 0.96 for the cheek, forehead, and abdomen skin models (Fig. 4).

## Discussion

We demonstrate a strong correlation between SC stresses measured by an ex vivo model system and in vivo tightness perception measured by several large human sensorial perception assessment surveys (Fig. 2g). The correlation is particularly relevant given that the surveys were conducted in 3 consecutive years and with study participants in different countries on two continents (Europe and Asia) compared to the ex vivo model studies that were conducted subsequently with tissue from donors on a third continent (North America). A further remarkable feature is that the correlation holds for both moisturizing treatments that decrease skin tightness and cleansers that elevate tightness. We found that the measured SC stresses were better related to tightness perception than other properties such as the measured SC water loss.

The strong correlation between SC stresses and tightness perception motivated the desire for a more fundamental understanding of the connection involving sensory neurons and neural pathways in the somatosensory system. It is not obvious that the relation between measured stresses and skin tightness perception scores would indicate a correspondingly strong relation between the perception scores and predicted neural firing rates given the nonlinearities in the skin layer mechanical properties and geometry. We were interested in investigating the mechanism that gives rise to skin tightness perception by modeling how the SC stresses changed the skin mechanical environment to activate mechanoreceptors and modulate neural signals, which ultimately are correlated to the perception of skin tightness. Using the full thickness skin FE simulations of treatment-dependent skin strains and our SAI firing rate model adapted from literature-reported primate studies, we found a strong correlation between the mean computed firing rate among elements near the DEJ in FE simulations and skin tightness perception scores.

The mean firing rate calculations provide a link for the correlation between measured SC drying stresses and tightness perception scores of the skin treatments. We found that a firing rate model dependent on the effective strains computed in FE simulations are adequate predictors of SAI neural activity, capturing the concept that multiple modes of deformation of Merkel cells can open ion channels and elicit a response in the afferent neuron. The mean SAI afferent firing rate is used in relations with tightness perception since mean neural response can provide for stronger correlation between neural activity and perception than the response of a single afferent (74).

Our firing rate model averages the firing rates calculated as if SAI afferent endings could be located anywhere along the epidermal side of the DEJ, removing the need to assume the precise locations of SAI afferent neuron endings when evaluating computed strains. Our finding that tightness perception scores and mean computed SAI afferent firing rates are linearly related aligns with the postulation in psychophysics that perception and neural activity are linearly related (75).

It is interesting to note that participants were capable of detecting skin tightness after the hours-long process of SC drying following the application of a treatment. As it is unlikely that they constantly perceived skin tightness while SC stresses slowly increased, we can assume that participants habituated to the stimulus of SC drying. When evaluating a treatment, study participants’ deliberate attention to regions of the skin where they applied the treatment would have prompted a conscious awareness of tightness despite the gradual and imperceptible accumulation of significant stresses in the SC. It is also possible that movement of the dried skin, for example, from a facial gesture, could stimulate firing of fast-adapting neurons, and this transient neural response could contribute to bringing the stimulus of dried SC to the conscious awareness. Such fast-adapting neural activity is expected to be transient, with a duration that is shorter than the sustained conscious perception of skin tightness. Though the integration of both fast-adapting and slowly adapting neural responses may contribute to the conscious perception of skin tightness, this work has focused on the role of SAI neurons in the sustained perception of skin tightness. Future studies could investigate the effects of dried SC on the relatively transient fast-adapting neural responses.

In developing the model relating strain to SAI neuron firing rates, we relied on neural data from a primate fingertip (67). While there is evidence that SAI neural responses are similar between humans and primates (75), we assume that the relation between strains and firing rates is valid regardless of the location of SAI afferents in the body. Variability in the response to identical stimuli between neurons is observed (67). Thus, there is uncertainty in the equation that relates strain and SAI afferent neuron firing rates. It remains noteworthy, however, that such a strong linear trend is observed between predicted mean SAI afferent firing rates and reported tightness perception scores.

Our study did not seek to establish a minimal perceptible change in firing rates but rather to demonstrate the clear connection between firing rates computed using detailed biomechanical characterization as inputs to the firing rate model constructed from previously established mechanoreceptor activation. The firing rates predicted clearly correlate strongly with the skin tightness perception scores from a large group of subjects indicating that the subjects could distinguish differences between the treatments that in the present study ranged from ∼2 to ∼6 imp/s. In the context of our study and firing rate model, a 1-imp/s change does appear to be perceptible. We hope, however, that the present study will encourage future neurological and psychophysical studies that reveal the minimum perceptible change in firing rates. This would include aspects of habituation and the number of mechanoreceptors being activated, since for large skin surfaces like on the face, many mechanoreceptors would be simultaneously activated amplifying the neurological signaling.

Topography of the skin is responsible for driving strain from the SC into the lower skin layers (compare simulations in Fig. 1d for flat and uneven topography). By modeling multiple real skin surface topographies, we observe the varied effect of topography on strain fields in underlying skin from SC contraction. We modeled sections of skin from the cheek and forehead, consistent with the evaluation of skin tightness perception by participants in the perception assessment survey after application of topical treatments to facial skin. Additionally, we modeled a third abdominal skin section to contribute to the diversity of skin morphologies studied. The correlations between mean computed firing rates and tightness perception scores are strongly linear for all three skin sections analyzed. The results from three skin section models support the robustness of our methods and findings, as the results are not a coincidental effect of a specific skin topography. Future work highlighting the sensitivity of SAI afferent responses to variations in skin surface topography, skin layer mechanical properties, and skin layer thicknesses due to characteristics subject to biovariability such as age could enhance insight into which factors influence the acuity and strength of skin tightness perception. In addition, direct validation of the work could be attempted using microneurography in humans to directly measure SAI firing rates under different biomechanical conditions while simultaneously recording perceptual feedback to the different biomechanical conditions. While great care was taken to avoid subjectivity in the survey data collection, the authors acknowledge that an objective measure of mechanoreceptor activity would further bolster this work.

Bias in the design of the perception assessment survey for evaluating the cleansers and moisturizers did not significantly compromise interpretation of the results. A clear differentiation in the effect of moisturizing and cleanser treatments on perceived comfort was observed. The in vivo human perception assessment study timescale (∼12 hours) coincided well with the measured in vitro drying stress profiles (8–12 hours). There did not appear to be a bias between the perceived magnitudes of tightness for a stress increasing or stress decreasing formula. That is, survey results suggest a similar relative change in tightness perception score for a 10% reduction in SC stress as for a 10% increase in SC stress. The 2–10-MPa values for biaxial stress in the SC are sufficient to trigger neural responses without the firing rates reaching a plateau at higher drying stresses, which supports the studied treatments being perceived distinctly with respect to skin tightness. Inherent sources of error such as subjectivity and language differences (French and Chinese) between the two surveys do not seem to compromise the robustness of these results.

Due to the complexity of each formulation, the role of each component in the formulations is not explored in this study. It has been previously shown, though, that individual components can have marked effects on SC drying stresses (76). Further work in the area of formulation chemistry may reveal the combinatory effect of several key ingredients common or dissimilar between the treatments explored in this study.

The approach of this study can be used to inform the design of skin treatments such as moisturizers and cleansers as it elucidates a means of predicting the efficacy of skin treatments for minimizing stresses in the skin and maximizing skin comfort. In vivo, drying skin can lead to significant discomfort, manifested as a perception of skin tightness, in addition to driving damage processes. Understanding how to reduce stresses via the application of skin treatments will make the treatments more desirable and mitigate skin damage in the form of cracking, which introduces a pathway for infection. The process of studying the effects of skin treatments in ex vivo experiments and in numerical simulations can also reduce dependence on large human sensorial perception assessment trials to evaluate novel treatment formulations.

Additionally, this work provides further quantitative insight into the mechanics of mechanoreceptor activation of interest in neuroscience and wearable electronics. For example, the aim to communicate passively through the skin is mediated by contact with and deformation of the outer SC layer, which influences deformations at depths of the skin with high mechanoreceptor density. This study lays the groundwork for the understanding of how SC deformations are transmitted and perceived by SAI neurons.

## Conclusion

A quantitative biomechanical mechanism is described whereby cutaneous mechanoreceptors and corresponding sensory neurons are distinctly activated in response to an array of topical skin treatments. The resulting neural signaling is used to explain the skin sensory perception subjectively reported by the perception assessment participants in a comprehensive study involving over 2,000 women in different countries over a 3-year period. A FE model of human skin from three anatomical sites was used to compute detailed strain distributions in full-thickness human skin. A separate FE model was developed for predicting SAI afferent firing rates based on strain induced from indentation contact in primate fingertips. It was subsequently used to predict mean firing rates among SAI afferent neurons in full-thickness skin at the three anatomical sites in response to SC stresses that developed from several topical skin treatments. Strong correlations are observed between skin sensory perception survey scores and the computed SAI neuron firing rates in full-thickness skin, implying that SAI neurons contribute to the perception of biomechanical changes of skin, such as skin tightness. The combination of experimental measurements and computational modeling presented in this study elucidates the biomechanical mechanism by which skin sensorial perception arises from the activation of cutaneous SAI neurons following the use of topical skin treatments.

## Data Availability

Original data created for the study are or will be available in a persistent repository upon publication.
